# Antinociceptive and Abuse Potential Effects of Cannabinoid/Opioid Combinations in a Chronic Pain Model in Rats

**DOI:** 10.3390/brainsci9110328

**Published:** 2019-11-17

**Authors:** Mohammad Alsalem, Ahmad Altarifi, Mansour Haddad, Sara A. Aldossary, Heba Kalbouneh, Nour Aldaoud, Tareq Saleh, Khalid El-Salem

**Affiliations:** 1 Faculty of Medicine, The University of Jordan, Amman 11942, Jordan; heba.kalbouneh@gmail.com (H.K.); nour.aldaoud@hotmail.com (N.A.); 2 Faculty of Medicine, Jordan University of Science and Technology, Irbid 22110, Jordan; aaaltarifi@just.edu.jo (A.A.); khalidelsalem@hotmail.com (K.E.-S.); 3 Faculty of Pharmacy, Philadelphia University, Amman 19392, Jordan; dr.man.haddad@gmail.com; 4 Faculty of Clinical Pharmacy, King Faisal University, Hofuf 31982, Saudi Arabia; saldossary@kfu.edu.sa; 5 Faculty of Medicine, The Hashemite University, Zarqa 13133, Jordan; saleht2@mymail.vcu.edu

**Keywords:** chronic pain, complete Freund’s adjuvant (CFA), von Frey, morphine, CP55940, tramadol, intracranial self-stimulation (ICSS)

## Abstract

Chronic pain is a persistent and debilitating health problem. Although the use of analgesics such as opioids is useful in mitigating pain, their prolonged use is associated with unwanted effects including abuse liability. This study assesses the antinociceptive effect of combining subtherapeutic doses of two opioids (morphine or tramadol) with the synthetic cannabinoid CP55940 (2-[(1R,2R,5R)-5-hydroxy-2-(3-hydroxypropyl)cyclohexyl]-5-(2-methyloctan -2-yl)phenol). It also evaluates the associated adverse effects of these drugs and combinations. Adult male rats were injected with intraplantar complete Freund’s adjuvant (CFA) to produce mechanical allodyia. Antinociceptive effect of morphine, tramadol, the synthetic cannabinoid CP55940, or their combinations was evaluated three to nine days post-CFA injections. Intracranial self-stimulation (ICSS) was utilized to evaluate the abuse liability of these drugs or their combinations. All drugs alone produced a dose-dependent antinociceptive effect. Morphine produced minimal effect on ICSS, but both tramadol and CP55940 produced dose-dependent depression of ICSS. Morphine at a dose of 0.32 mg/kg enhanced the antinociceptive effects of CP55940, in that, CP55940 produced antinociception at a lower dose (0.1 mg/kg) when compared to the vehicle. The aforementioned combinations did not change CP55940-induced depression of ICSS. On the other hand, tramadol failed to enhance the antinociceptive effect of CP55940. Our data suggest that combining CP55940 with morphine, but not tramadol, shows a better antinociceptive profile with no additional risk of abuse liability, which represents a potential pain management approach.

## 1. Introduction

Opioids such as morphine are potent and widely used analgesics. They are considered to be the drugs of choice for the treatment of moderate to severe pain [1]. However, the use of opioids has been limited due to their unfavorable effects such as abuse liability [2]. Thus, the analgesic potential of other compounds such as cannabinoids is being investigated for the clinical treatment of miscellaneous forms of pain, particularly where conventional analgesics (e.g., opioids, tramadol, non-steroid anti-inflammatory drugs) have lower efficacy. This becomes especially crucial in certain forms of inflammatory, neuropathic, or chronic pain conditions [3,4,5]. In this regard, while preclinical studies have shown that several cannabinoid agonists have antinociceptive effects against both acute and chronic pain, clinical studies indicate that cannabinoids are more effective in the management of chronic pain [5].

Cannabinoid signaling is largely involved in the regulation of nociception, representing a fundamental target for pharmacological intervention [3,6]. Most cannabinoid receptor ligands such as CP55940 (2-[(1R,2R,5R)-5-hydroxy-2-(3-hydroxypropyl)cyclohexyl]-5-(2-methyloctan-2-yl) -phenol) act preferentially through the activation of the cannabinoid receptor 1 (CB1) receptor [7]. The activation of CB1 produced antinociceptive effects in several laboratory species using several methods to analyze pain sensitivity in both acute and chronic pain models [6,8,9]. This was confirmed by the ability of CB1 receptor antagonists to also produce hyperalgesia [10]. Moreover, CB1 receptor knockout mice revealed substantial alterations in pain sensitivity when compared to their wild-type counterparts [11]. However, cannabinoid-induced analgesia is not completely absent in CB1-knockout mice, suggesting a potential contribution of other pharmacological targets such as CB2 receptors [12]. Key central structures (both supraspinal and spinal) are implicated in nociception regulation, in addition to the peripheral sensory nerve terminals (which also express CB2 receptors) [12,13,14]. All of the aforementioned experimental evidence supports that the endocannabinoid pathway is heavily involved in nociceptive control, and a potential alternative for pharmacological modulation.

Pharmacological additive or synergistic approaches have been utilized to enhance the analgesic effects of several compounds, especially when using lower doses can lower the risk of undesirable side effects [7]. In this context, the opioid and cannabinoid antinociceptive systems largely overlap. This has been confirmed in several pharmacological, biochemical, and anatomical studies [15,16,17]. For example, the combination of CP55940 and morphine produced greater antinociceptive effect than morphine alone in assays of pain-elicited behavior [17,18,19,20]. Moreover, low doses of cannabinoid receptor agonists such as CP55940 enhanced the antinociceptive potency of sub-effective doses of morphine in various nociceptive assays in rodents [21]. Furthermore, it has been previously shown that administering a combination of cannabinoids and opioids (and other pain-relieving medications) at sub-effective doses may produce more antinociceptive responses when compared to individual treatments at higher doses in preclinical studies [15,22]. These examples are potentially associated with less frequent adverse effects resulting from prolonged use of high-dose opioids [23,24,25]. In particular, the abuse-related effects of opioids were not increased by coadministration of cannabinoids in monkeys [16,26]. All of these studies support the conclusion that such interactions may have important therapeutic applications.

Given the clinical need for effective and safe treatments for pain, the aim of this study was to determine the antinociceptive effects and abuse liability of two opioids (morphine and tramadol) in combination with the CB1 receptor agonist, CP55940, using von Frey behavioral and intracranial self-stimulation (ICSS) behavioral assays, respectively. Tramadol is a synthetic opioid that exhibits both mild opioid receptor-binding and norepinephrine and serotonin reuptake inhibition [27,28]. It is generally well tolerated in the treatment of pain and causes few opioid side effects.

## 2. Materials and Methods

### 2.1. Evaluation of the Effects of Drugs/Combinations on the Inflammatory Pain Model

#### 2.1.1. Animals

Behavioral experiments were done on adult male Sprague-Dawley rats (200–250 g). The experiments were conducted at the University of Jordan laboratories. Rats were collectively sheltered at the University of Jordan Animal House Unit in a temperature controlled environment 22 ± 1 °C at a 12 h:12 h light:dark cycle. Methods were approved by the scientific research committee at the University of Jordan. Procedures were carried out in agreement with the Animal (Scientific Procedure) Act 1986 and International Association for the Study of Pain guidelines.

#### 2.1.2. Induction of Inflammatory Pain Model

Initially, baseline nociceptive thresholds were measured for three days. Afterward, intraplantar injections of either the vehicle or complete Freund’s adjuvant (CFA; 50% in saline, with 5 mg/mL heat-killed *mycobacterium tuberculosis*, 0.1 mL) were administered in the left hindpaw of each rat in order to induce inflammatory pain. Consequently, rats were experimented on every other day for nine days following the CFA injection [29].

#### 2.1.3. Assessment of Mechanical Allodynia

Von Frey filaments were used to measure responsiveness to pressure stimulus. First, rats were individually placed in plastic cages. These cages provide full access to the rats’ hindpaws as they have a wire mesh bottom. Behavioral accommodation was permitted for each rat for at least 25 min until cage inspection and main grooming actions finished. After that, von Frey filaments were used according to the “up–down” method (2–15 g, with logarithmically incremental stiffness; Bioseb, Vitrolles, France) to the mid-plantar surface of the left hind paw of each rat. During each time, the von Frey hair was held perpendicularly to the paw’s planter aspect for about 6–8 s [30]. The withdrawal threshold data were determined in grams as paw withdrawal thresholds (PWT).

The effects of different drugs or combinations on CFA-induced nociceptive behavior were assessed at days 3–9 post-CFA injection using a Latin square design. CP55940 (0.032, 0.1, and 0.32 mg/kg), morphine (0.32, 1, and 3.2 mg/kg) and tramadol (1, 3.2, and 10 mg/kg) were introduced through intra-peritoneal (I.P.) injections (0.5 mL, *n* = 6 rats/group). The von Frey filament test was performed 30 min after drug/combination administration.

#### 2.1.4. Data Analysis

A Latin square design was used in conducting these experiments. CFA-induced mechanical allodynia were first measured as PWT in grams, and then the antinociceptive effects of different drugs or combinations were presented as means ± SEM% antinociception. Percent antinociception was calculated according to the following equation:

(1) $$\%\;\mathrm{Antinociception}\;=\;\frac{\mathrm{PWT}\;\mathrm{after}\;\mathrm{drug}\;\mathrm{application}\;-\;\mathrm{PWT}\;\mathrm{before}\;\mathrm{drug}\;\mathrm{application}}{\mathrm{baseline}\;\mathrm{PWT}-\;\mathrm{PWT}\;\mathrm{before}\;\mathrm{drug}\;\mathrm{application}}.$$

Two-way ANOVA analysis followed by Holm–Sidak post hoc was used with treatment and time as the main factors. Additionally, Dunnett’s post-hoc test was used when appropriate following one-way ANOVA test. Statistical analysis used the Graph Pad statistical program (Prism 6, San Diego, CA, USA).

### 2.2. Assay of Intracranial Self-Stimulation

#### 2.2.1. Animals

Adult male Sprague-Dawley rats (Harlan, Indianapolis, IN, USA) weighed approximately 300 to 320 g upon arrival at the facility. Each subject was housed individually and maintained on a 12-h light/dark cycle (lights on from 6:00 to 18:00). Subjects had free access to food and water ad libitum (except during testing). Animal maintenance and research were in compliance with the National Institute of Health’s Guide for the Use and Care of Laboratory Animals (Institute of Laboratory Animal Resources, 1996) and adhered to the guidelines of the Committee for Research (National Research Council, 2003) and Ethical Issues of the International Association for the Study of Pain. All animal use protocols were approved by the Virginia Commonwealth University and Jordan University of Science and Technology Institutional Animal Care and Use Committees.

#### 2.2.2. Surgery

All rats were anesthetized using isoflurane (2.5–3% in oxygen; Webster Veterinary, Phoenix, AZ, USA) to implant stainless-steel electrodes (Plastics One, Roanoke, VA, USA). One pole (the cathode) of each bipolar electrode was 0.25 mm in diameter and covered with polyamide insulation except at the flattened tip, and the other pole (the anode) was 0.125 mm in diameter and uninsulated. The cathode part of the electrode was implanted in the left medial forebrain bundle at the following coordinates: 2.8 mm posterior to bregma, 1.7 mm lateral to the midsagittal suture, and 8.8 mm below the surface of the skull. The anode was wrapped around one of the three skull screws to serve as the ground. The electrode and the screws were fixed to the skull with orthodontic resin. The animals were allowed to recover for seven days before proceeding to behavioral training.

#### 2.2.3. Apparatus

Experiments were performed in sound-attenuating boxes that contain modular acrylic test chambers (29.2 × 30.5 × 24.1 cm). Each box is equipped with a response lever, stimulation lights above the response lever, a 2-W house light, and an ICSS stimulator (MED Associates, St. Albans, VT, USA). Electrodes were connected to the stimulator via a swivel connector (model SL2C; Plastics One, Roanoke, VA, USA). The stimulator was controlled by computer software that also controlled the programming and data collection (MED Associates, St. Albans, VT, USA).

#### 2.2.4. Behavioral Procedure

Animal behavioral testing and data analysis were similar to those described previously [2,31]. Initially, animals were trained for free lever pressing for at least three days. Each lever press produced a delivery of a 0.5 s train with a 0.1-ms pulse duration, which was accompanied by the illumination of the stimulus lights. Any pressed lever during the 0.5-s stimulation period did not produce additional stimulation. At the beginning, the frequency of stimulation was held constant at 158 Hz, and the stimulation intensity for each rat was modified gradually to the lowest value that would maintain a the rate of reinforcement above 30 stimulations per minute. This intensity (120–250 μA across rats) was then held constant for the remainder of the study before the frequency manipulations were introduced. Each frequency session consisted of multiple 10-min components. During each component, a descending series of 10 frequencies (158–56 Hz in 0.05-log increments) was presented, with each frequency available during sequential 1-min frequency trials. Each frequency trial began with a 10-s timeout, during which responding had no scheduled outcomes. During the last 5 s of this timeout, five non-contingent stimulations were delivered at the frequency available during that trial, and the lever lights were illuminated. Afterward, there was a 50-s “response” phase, during which each lever press produced electrical stimulation under the continuous reinforcement schedule. Training continued with the presentation of two to three sequential components per day until the rats reliably responded for the first four to six frequency trials of all components for at least three consecutive days.

Once training was completed, testing was initiated. The first component of each test session was considered the acclimation period and the data were not included in the statistical analysis. Only the data from the second and third “baseline” components were used to calculate the baseline parameters of the total stimulations for that session (see Data Analysis). Drugs were injected immediately after the end of the third baseline component, and each subject remained in its assigned test chamber. The effects of CP55940, morphine, or tramadol were studied in a group of five rats. Subjects were kept in the operant chambers after the administration of CP55940 (0.032–0.32 mg/kg; s.c.), morphine (0.32–3.2 mg/kg; i.p.), tramadol (1.0–10 mg/kg; i.p.), or their vehicles. After 30 min, two more consecutive “test” components were conducted, totaling 20 min. In the next phase of the study, the same protocol and pretreatment time were used to test the effect of selected doses of morphine or tramadol (i.p.) co-administered with CP55940 (0.032–0.32 mg/kg; s.c.).

#### 2.2.5. Data Analysis

The primary dependent variable in this ICSS procedure was the reinforcement rate in stimulations per minute during each frequency trial. The total number of stimulations per component was calculated as the sum of stimulations delivered across all 10 frequency trials of each component. Test data were then normalized to individual baseline data by using the equation percentage of baseline total stimulations per component = (mean total stimulations per test component ÷ mean total stimulations per baseline component) × 100%. Data were then averaged across rats in each experimental condition and compared by repeated-measures one-way ANOVA. A significant one-way ANOVA was followed by Dunnett’s post hoc test, a significant two-way ANOVA was followed by the Holm–Sidak post hoc test, and the criterion for significance was set at *p* < 0.05.

## 3. Results

### 3.1. Effects of Test Drugs on Inflammatory Pain Model

A few hours following the injection of CFA into the plantar aspect of the hindpaw, notable local inflammatory signs such as erythema and edema were observed. These findings are in agreement with previous studies that have demonstrated the ability of CFA to induce inflammatory pain [32]. The PWT was significantly reduced when compared to baseline measures as early as day 1 post-injection and lasted until day 19 (data not shown).

**Figure 1:**
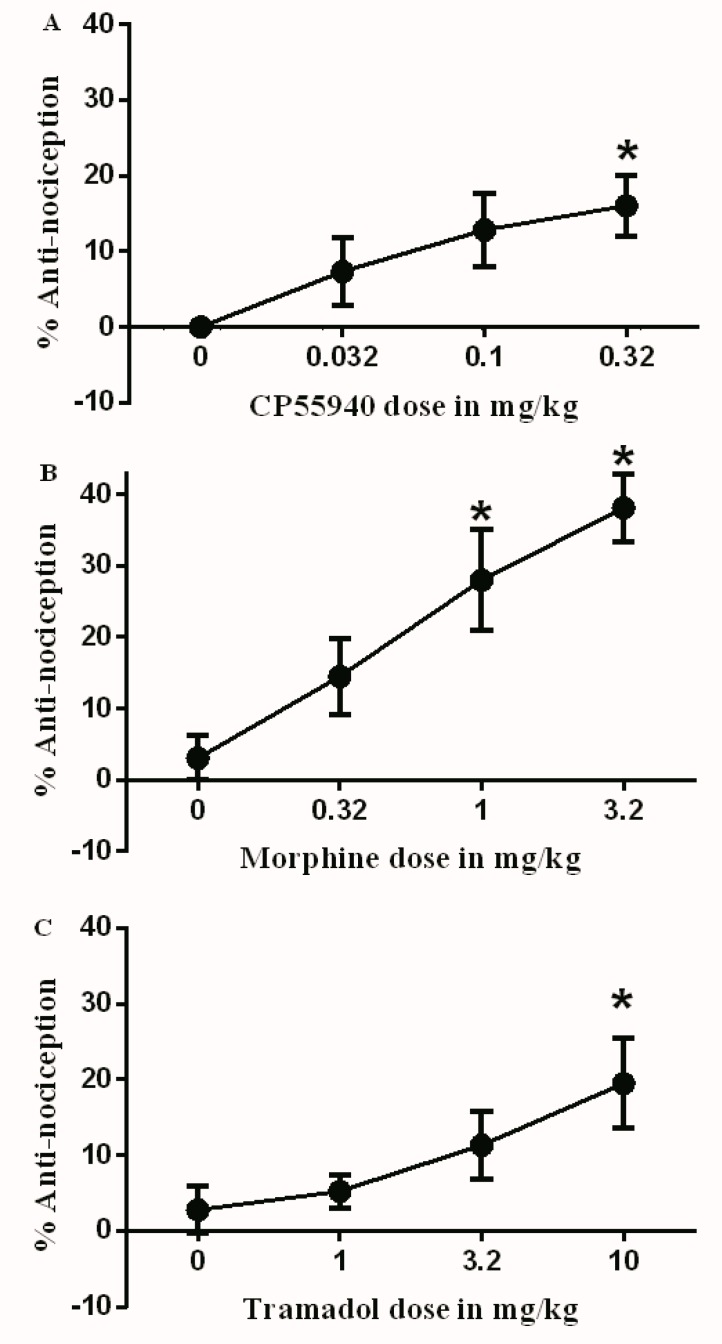
Antinociceptive effects of (**A**) CP55940 (0.032, 0.1, and 0.32 mg/kg); (**B**) morphine (0.32, 1, and 3.2 mg/kg); and (**C**) tramadol (1, 3.2, and 10 mg/kg) on complete Freund’s adjuvant (CFA)-induced mechanical allodynia. Data are presented as the mean ± SEM of %antinociception. %antinociception = (paw withdrawal thresholds (PWT) Post-drug – PWT Pre-drug)/(baseline PWT – PWT pre-drug). Data were analyzed using the one way ANOVA test followed by Dunnett’s post hoc test (* *p* < 0.05, *n* = 6 rats per group).

On day 3 following CFA injection, multiple doses of different drugs were administered: vehicle (3% Tween 20 in saline), CP55940 (0.032, 0.1, and 0.32 mg/kg), morphine (0.32, 1, and 3.2 mg/kg), and tramadol (1, 3.2, and 10 mg/kg). After 30 min, the antinociceptive effects of these drugs were evaluated. CP55940 produced a significant antinociceptive effect only at the highest dose (0.32 mg/kg) compared to the vehicle (Figure 1A). Additionally, morphine and tramadol produced a significant antinociceptive effect when compared to the vehicle at 1–3.2 mg/kg and 10 mg/kg, respectively (Figure 1B,C).

**Figure 2:**
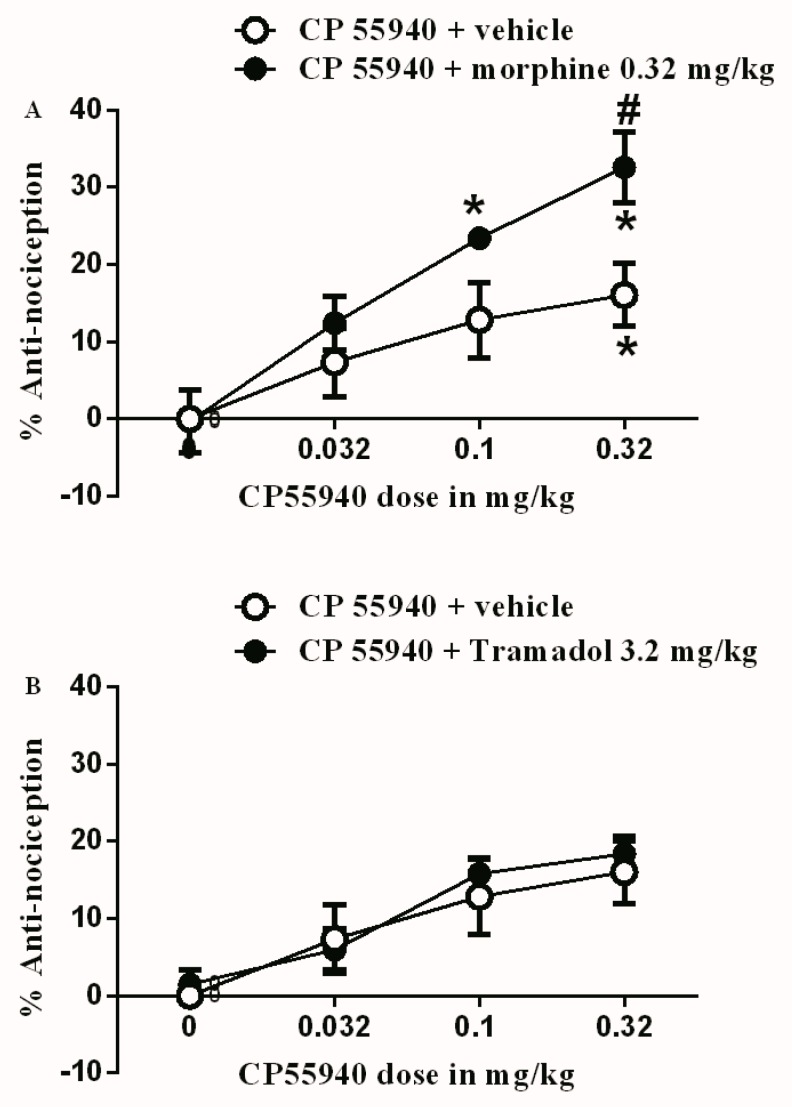
(**A**) Antinociceptive effects of CP55940 (0.032, 0.1 and 0.32 mg/kg) either with vehicle or with sub-therapeutic dose of morphine (0.32 mg/kg) on CFA-induced mechanical allodynia. (**B**) Antinociceptive effects of CP55940 (0.032, 0.1, and 0.32 mg/kg) either with vehicle or with sub-therapeutic dose of tramadol (3.2 mg/kg) on CFA-induced mechanical allodynia. Data are presented as the mean ± SEM of %antinociception. %antinociception = (PWT Post-drug – PWT Pre-drug)/(baseline PWT – PWT pre-drug). Data were analyzed using two-way ANOVA followed by the Holm–Sidak post hoc test, ^#^ indicates a significant difference between rat groups. * indicates a significant difference in comparison with day 0 within each rat group.

The subtherapeutic dose of morphine (0.32 mg/kg) was co-administered with the different doses of CP55940 (0.032, 0.1, and 0.32 mg/kg). The 0.32 mg/kg morphine dose was able to enhance the antinociceptive effect of CP55940. In other words, CP55940 produced antinociception at a lower dose (0.1 mg/kg) when compared to the vehicle (Figure 2A). Additionally, the antinociceptive effect of 0.32 mg/kg CP55940 was enhanced by the co-administration of 0.32 mg/kg morphine. In contrast, the subtherapeutic dose of tramadol (3.2 mg/kg), when co-administered with the different doses of CP55940, did not produce any change to the antinociceptive effects of CP55940 (Figure 2B).

### 3.2. Effects of Test Drugs on Intracranial Self-Stimulation

**Figure 3:**
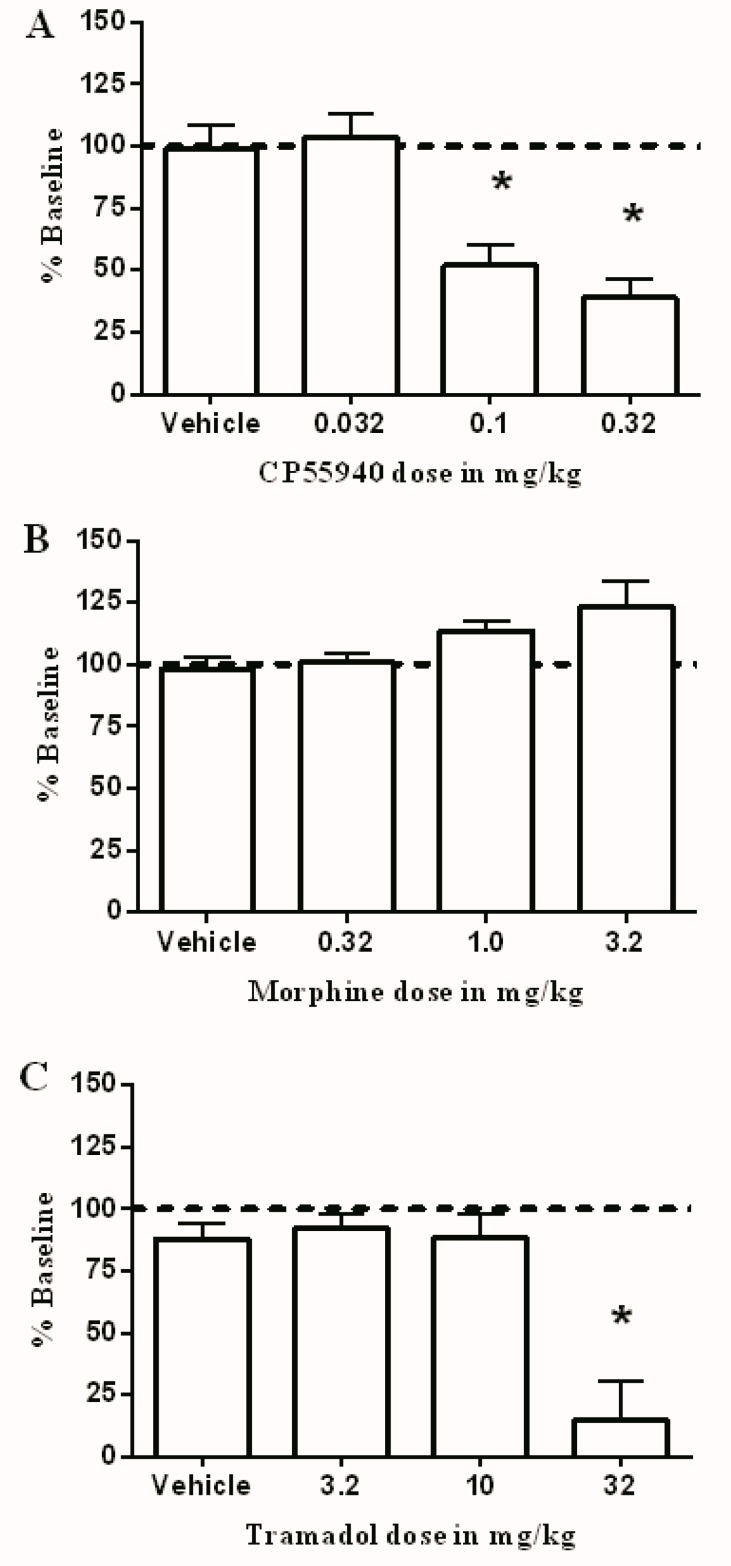
Effect of CP55940 (**A**), morphine (**B**), or tramadol (**C**) and their combination on intracranial self-stimulation in rats. Abscissae: dose of drug. Ordinates: percent baseline stimulations per test component. Data were analyzed using the one way ANOVA test followed by Dunnett’s post hoc test (* *p* < 0.05, *n* = 6 rats per group).

The average intensity ± SEM for all rats was 148 ± 25.15 μA. Figure 3A shows that CP55940 produced dose-dependent depression of ICSS. This depression was significant after 0.1 and 0.32 mg/kg. On the other hand, morphine did not produce a significant increase in ICSS at the tested doses (Figure 3B). Additionally, like CP55940, tramadol produced a decrease in ICSS, which was significant after 10 mg/kg when compared to the vehicle (Figure 3C).

**Figure 4:**
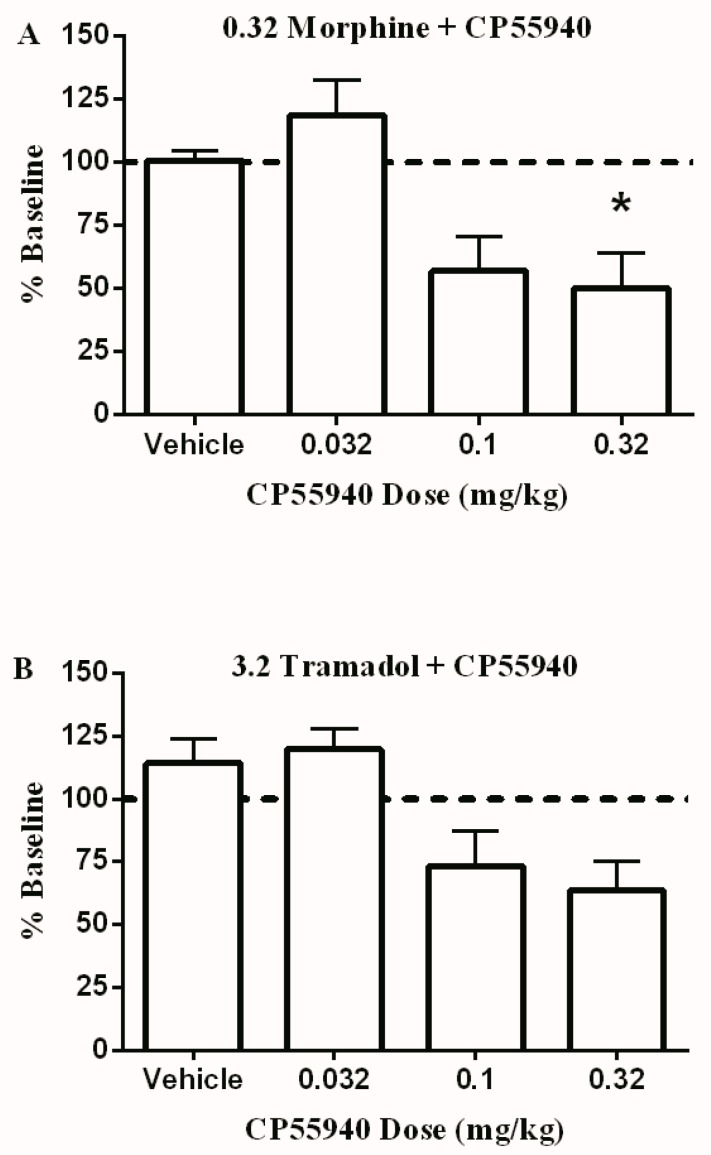
The effect of different doses of CP55940 on ICSS in combination with 0.32 mg/kg morphine (**A**) or 3.2 mg/kg tramadol (**B**). Abscissae: dose of CP55940. Ordinates: percent baseline stimulations per test component. Data were analyzed using the one way ANOVA test followed by Dunnett’s post hoc test (* *p* < 0.05, *n* = 6 rats per group).

Finally, Figure 4 shows the effect of co-administering the same doses of CP55940 with 0.32 mg/kg morphine (Figure 4A) or 3.2 mg/kg tramadol (Figure 4B). In both cases, CP55940 produced a dose-dependent decrease in total brain stimulations; however, this decrease was only significant when 0.32 mg/kg CP55940 was co-administered with 0.32 mg/kg morphine.

## 4. Discussion

This study examined the antinociceptive effect and abuse potential of the CB1 agonist CP55940 in combination with two commonly used opioids: morphine or tramadol. There were four major findings in this work. First, each of the tested drugs produced a partial dose-dependent antinociceptive effect on CFA-induced mechanical allodynia. Second, the co-administration of a subtherapeutic dose of morphine, but not tramadol, enhanced the antinociceptive effect of CP55940 in the inflammatory pain model. Third, CP55940 and tramadol, but not morphine, produced a dose-dependent decrease in ICSS. Finally, the administration of subtherapeutic doses of either morphine or tramadol attenuated the CP55940-induced depression of ICSS. Collectively, these data suggest that combining cannabinoid agonists with subtherapeutic doses of opioids may enhance their antinociceptive effects without increasing the risk of abuse liability.

The primary finding of this study showed that the nociceptive process was decreased in a dose-dependent manner by individual injections of CP55940, morphine, or tramadol. The antinociceptive effect of both morphine and tramadol on CFA-induced mechanical allodynia is consistent with previous findings [33,34,35,36]. However, both drugs did not produce a complete reversal of the mechanical threshold at the tested doses. Other studies showed a complete blockade of CFA-induced mechanical allodynia at higher morphine and tramadol doses, but was associated with other behavioral changes such as sedation [37]. Since the aim of the current study was to examine subtherapeutic doses of both drugs in combination with the CB1 agonist, higher doses were not tested. While a previous study showed that CP55940 was able to reduce the spinal nociceptive transmission induced by CFA through a CB1-dependent mechanism, to our knowledge, this is the first study to examine the effect of CP55940 on CFA-induced mechanical allodynia [38]. It is noteworthy that other CB1 agonists produced robust antinociception in the CFA inflammatory model in agreement with our findings [39,40].

Results from the current study suggested that CP55940 possibly produces sedation and an impairment of motor coordination in addition to its antinociceptive effects. In previous studies, this effect produced by CP55940 was found to be abolished in CB1 (but not CB2) receptor knockout mice [41]. Therefore, the results in the present study suggest that the antinociceptive effects of CP55940 are more likely to be mediated by CB1 receptors rather than CB2 receptors. However, it is also possible that the antinociceptive effects of CP55940 might be partially due to its anti-inflammatory effect mediated through the CB2 receptor signaling pathway [42]. Further research is required to investigate the underlying mechanisms of the antinociceptive effects of CP55940.

Morphine at a dose of 0.32 mg/kg was used in combination with CP55940 to measure their combined antinociceptive effect. This dose of morphine was selected because it did not produce a significant antinociceptive effect or significant increase in ICSS. The combination increased the potency and efficacy of CP55940′s antinociceptive effect. The synergistic/additive effect of administering both morphine and cannabinoids is well-documented in the literature in both rats and monkeys [16,17,43]. However, all of these studies tested this combination in acute pain models, and to our knowledge, this is the first study to test this combination in a chronic inflammatory pain model. Opioid agonists and exogenous cannabinoids couple to and activate G-protein coupled receptors and mediate common signaling pathways related to clinical issues of tolerance, dependence, abuse liability, and addiction [18].

Unexpectedly, the subtherapeutic dose of 3.2 mg/kg tramadol did not shift the dose-response curve of CP55940 in a similar manner to morphine’s action. One possible explanation of this observation is that the dose we selected as a subtherapeutic dose (3.2 mg/kg) was very small in a way that did not have a noticeable effect when combined with CP55940. However, this is less likely because 3.2 mg/kg tramadol alone produced 12% antinociception, which is equal to the antinociceptive effect produced by the used subtherapeutic dose of morphine (0.32 mg/kg morphine). Another possible explanation is that tramadol is not effective in enhancing CP55940-induced antinociception in chronic pain models. This explanation cannot be made entirely based on our data, and further studies are needed to confirm this conclusion. Similarly, based on our data, a double aversive effect of both substances (CP55940 and tramadol) is a valid possibility and needs further investigations.

The current study showed that CP55940 produced dose-dependent depression of ICSS in rats. Similar results have been previously reported in mice and rats [44,45]. In addition, other CB1 agonists produced similar effects in ICSS such as Δ9-tetrahydrocannabinol and WIN55,212-2 ((R)-(+)-[2,3-Dihydro-5-methyl-3-(4-morpholinylmethyl)pyrrolo[1,2,3-de]-1,4-benzoxazin-6-yl]-1-naphthalenylmethanone mesylate) [31,46]. This depression in ICSS can possibly be attributed to their sedative effect at high doses [7]. On the other hand, morphine’s effect on ICSS has been adequately studied and showed a variable action of increasing and decreasing ICSS at low doses and high doses, respectively [2,47,48]. However, this is the first study to report the effect of tramadol-induced depression of ICSS. Tramadol in the tested drugs produced a decrease in ICSS at the highest dose only. Other preclinical assays used to measure rewarding effects of drugs showed that tramadol produces drug self-administration and conditioned-place preference at low doses (1.0 mg/kg), which is indicative of its abuse potential [49,50]. Herein, tramadol depressed ICSS at only high doses, which is opposite to its abuse-related effects. This may be explained by the sedative effects produced by tramadol at higher doses as reported previously [51]. More studies are needed to elucidate the exact effect of tramadol on ICSS after acute and chronic administration.

More interestingly, the combination of CP55940 with either morphine or tramadol did not show any evidence of increasing ICSS. This may indicate that this combination is least likely to produce abuse potential in future clinical studies if subtherapeutic opioid doses are used. This is an important finding in this study, as one major challenge of using opioids in patients is the risk of abuse potential, which may consequently lead to opioid addiction.

In conclusion, treatment with the combination of CP55940 and morphine had an additive effect to decrease mechanical allodynia without enhancing their abuse potential effects. This suggests that the morphine–cannabinoid combination may have a greater relative impact on mechanical allodynia compared to their reinforcing side effects and potentially represents an improved treatment option for pain. Weak opioids such as tramadol may not be as effective as potent opioids when subtherapeutic doses are used in combination with cannabinoid agonists; however, further studies are needed to explain such differences. In addition, ICSS was performed on intact animals in this study. Future studies are required to evaluate the effects on animals with chronic pain conditions.
